# Production of Fungal Phytases from Agroindustrial Byproducts for Pig Diets

**DOI:** 10.1038/s41598-019-45720-z

**Published:** 2019-06-25

**Authors:** Elizabeth Bárbara Epalanga Pires, Anderson Junior de Freitas, Fernanda França e Souza, Rafael Locatelli Salgado, Valéria Monteze Guimarães, Francisco Alves Pereira, Monique Renon Eller

**Affiliations:** 1 Universidade Federal de Viçosa, Department of Food Technology; Laboratory of Biochemical and Fermentative Processes, Viçosa, MG Brazil; 2 Universidade Federal de Viçosa, Department of Biochemistry and Molecular Biology, Viçosa, MG Brazil; 3 Agroceres Multimix Nutrição Animal LTDA, Mato Grosso, Brazil

**Keywords:** Hydrolases, Biopolymers

## Abstract

The application of phytases for animal feed in developing countries is limited due to the high cost of these enzymes, determined by the importation fees and the expensive substrates used for their production. In this work, we have used agroindustrial byproducts for the production of extracts containing phytases, which were accessed for their stability focusing on the conditions found in the gastrointestinal tract of pigs. The fungus *Acremonim zeae* presented higher phytase production in medium containing cornmeal, while the yeast *Kluyveromyces marxianus* produced 10-fold more phytase when cultivated on rice bran. Process optimization increased the difference in productivity to more than 300 fold. The phytase from *A. zeae* was thermostable, with higher activity at neutral pH and 50 °C, but was inhibited at pH 2.5 and by various ions. The phytase activity in the *K. marxianus* extract was stable at a wide range of conditions, which indicates the presence of at least two enzymes. As far as we know, this manuscript describes for the first time the phytase production and the characteristics of the extracts produced by both these microbial species. These enzymes could be produced at low cost and have potential to replace enzymes currently imported for this purpose.

## Introduction

Feed for monogastric animals is formulated from legume and cereal seeds, mainly corn and soybean meal, which represents about 70% of the pig diet and substantially contributes to supply their energy, protein, mineral and vitamin needs. Most of the phosphorus in these seeds are found as phytate and are unavailable for monogastric animals^1,2^, reducing the absorption of cations and essential nutrients^3^. Feed supplementation with phosphorus increases production costs and may cause negative environmental impacts due to the excessive excretion of phosphorus by the animals through feces^4^. The addition of phytases provides the phosphorus and other nutrients in the feed, which reduces the need for supplementation^5^.

Although the use of phytases in feed is essential in animal breeding, the high cost of these enzymes limits their use, mainly in developing countries, due to the costs with importation and those associated to the substrates used for enzyme production. The substrates used as culture media for the producing microorganisms account for 30–40% of the final costs of enzyme production^6^. Agroindustrial byproducts have been increasingly used as alternative substrates for the production of phytases and other biomolecules^7^.

Thus, in this work, we search for new alternatives for the production of fungal extracts containing phytases with potential for application in pig diets. To our knowledge, this is the first manuscript to explore phytase production by a yeast from the genus *Kluyveromyces* using low cost media and to characterize extracts from *Kluyveromyces* and *Acremonium* for phytase activity.

## Material and Methods

### Selection of fungi producing phytases

*Aspergillus glaucus*, *A. japonicus*, *A. terreus*, *Acremonium zeae* A, *A. zeae* B, *A. zeae* C1, *Debaryomyces hansenii*, *Kluyveromyces marxianus*, *Penicillium chrysogenum*, *P. expansum*, *P. citrinum* were used and maintained in Potato Agar Dextrose (PDA). The microorganisms were incubated at 28 °C for seven days. The spores were recovered, diluted in 0.85% (w/v) saline solution, counted and maintained at −20 °C. The final concentration of 10^4^ spores mL^−1^ (filamentous fungi) or 10^6^ viable cells (yeast) mL^−1^ was used for inoculation.

Each microorganism was inoculated into the liquid culture medium [potato infusion (200 g L^−1^); sucrose (10 g L^−1^); peptone (5 g L^−1^); Tween-20 (2.5 mL L^−1^) and CaCl_2_ (0.10 g L^−1^)], pH 5.5 and incubated at 28 °C and 150 rpm^8^. After 72 h, the extracts were clarified by centrifugation (5000 *g* for 15 min) at 4 °C. The activity of phytases at the supernatants was evaluated, as described below^9^. The experiment was carried out in triplicate.

### Substrate selection

The filamentous fungi *A. zeae* B and the yeast *K. marxianus* were activated and inoculated in media containing rice, corn, wheat and soybean meal.

The fungus *A. zeae* B was cultivated via solid-state fermentation (SSF) in KH_2_PO_4_ (1.5 g L^−1^), MgSO_4_ (0.5 g L^−1^), CuSO_4_ (0.25 g L^−1^), ZnSO_4_ (10 mg L^−1^), NH_4_SO_4_ (2 g L^−1^), FeSO_4_ (18.5 mg L^−1^), KCl (50 mg L^−1^) and CaCl_2_ (1 g L^−1^) supplemented with 20% (m/v) of each substrate to be tested. The flasks were incubated at 28 °C for seven days. After fermentation, the supernatant containing the enzymes was recovered in 2% (m/v) CaCl_2_ solution, shaken for 2 h at 150 rpm, and centrifuged (5000 *g* for 15 min) at 4 °C. The supernatant was then evaluated for the phytase activity^9^.

The yeast *K. marxianus* was cultivated via submerged fermentation in YEPG medium (peptone 1% (m/v), 2% (w/v) glucose, and 0.5% (w/v) yeast extract), containing KCl (3 mg mL^−1^), ammonium sulfate (7.5 mg mL^−1^) and supplemented with 1% (w/v) of each substrate. It was incubated at 200 rpm and 28 °C for 48 h. The supernatant was recovered by centrifugation (5000 *g* for 15 min) at 4 °C and tested for enzyme activity^9^.

### Culture conditions for phytase production

The first condition tested for phytase production by the fungus *A. zeae* B and the yeast *K. marxianus* was the time of incubation, and the other conditions were sustained constant. For such, the extract of the fungus *A. zeae* B was evaluated for phytase activity after four, five, six, seven and eight days of incubation in medium containing 10% (m/v) corn meal, at 28 °C pH 5.0. The yeast was cultured in periods of one, two, three and four days of fermentation in medium containing 1% (m/v) corn meal at 28 °C, pH 6.4 and its extract was evaluated for phytase activity.

Next, both microorganisms were incubated at 20, 25, 30, 35, 40, 45 °C, for five (*A. zeae* B) or two (*K. marxianus*) days, in media containing 10% corn meal at pH 4.0 (*A. zeae* B) or 1% (m/v) rice bran, pH 5.8 (*K. marxianus*).

The medium pH was also evaluated. For such, the pH of the media was adjusted to 3.0, 4.0, 5.0 and 6.0. The filamentous fungi were cultivated for five days at 28 °C in solid state fermentation using 10% (m/v) of corn meal and the yeast for two days at 28 °C in 1% (m/v) rice bran, and their extracts were evaluated for phytase activity.

Finally, different substrate concentrations were individually tested for the composition of the production media. Corn meal was added at 20, 30, 40 and 50% (m/v) for phytase production by *A. zeae* B, and rice bran at 1, 2, 3 and 4% (w/v) by the yeast *K. marxianus*. *A. zeae* B was cultivated for 7 d and the yeast, for 24 h, both at 28 °C.

### Phytase activity in the extracts

The extracts produced by the fungus *A. zeae* B and the yeast *K. marxianus* were characterized for the extrinsic conditions that favor phytase activity or stability, as follows:

#### Effect of pH on phytase activity

The extracts were incubated for 1 h at 4 °C in media with pH adjusted to 2.5 and 4.5 (0.1 M sodium citrate buffer); 5.0 and 6.5 (0.1 M sodium acetate buffer) and 7.0 and 10.0 (0.1 M Tris-HCl buffer). Then, the extracts were subjected to the phytase activity assay using buffers with pH values adjusted accordingly, respectively.

#### Effect of temperature on phytase activity

Phytase activity was evaluated at 10, 20, 30, 40, 50, 60, 70 and 80 °C in each extract.

#### Thermostability of the phytases in the extracts

The enzymatic extracts were diluted in Tris-HCl 0.1 M buffer pH 7.5 and incubated at 80 and 90 °C. The aliquots were withdrawn at 15, 30, 60 and 80 min, and the residual activity was evaluated.

#### Effect of metal ions on phytase activity

The effect of CaCl_2_, CaCO_3_, AgSO_4_, MgCl_2_, ZnSO_4_, CuSO_4_, CaSO_4_, NH_4_Cl at 2 and 5 mM on phytase activity was evaluated in the extracts at 50 °C.

The results of thermostability and effect of ions were expressed as the relative activity, considering the initial activity as 100%. The tests were performed in triplicate.

### Activity assay of phytase

The phytase activity was determined according to^9^ with modifications using 200 μL of 5 mM sodium phytate added to 100 mM sodium acetate buffer (pH 5.0) and 150 μL of the extract. The reaction was performed in water bath at 50 °C for 30 min, quenched with 250 μL of 10% (v/v) trichloroacetic acid solution. Then, 1000 μL of the colorimetric reagent, prepared from 10% (w/v) ammonium molybdate solution in 5 M sulfuric acid solution, were added to the test tubes. The reagent was prepared at the time of use by mixing 10% (v/v) of the solution to 5% (w/v) ferrous sulfate and deionized water. The absorbance was measured at 700 nm. The absorbance values were correlated with a standard curve of KH_2_PO_4_. The enzymatic activity analyses were performed in triplicate. An enzyme unit (U) was defined as the amount of enzyme required to release 1 μmol of inorganic phosphate per minute under the assay conditions.

### Statistical analysis

The data were compared using the GraphPad PRISM software system (version 6.0) by ANOVA, followed by the Tukey test (P < 0.05).

## Results and Discussion

### Selection of phytase-producing microorganisms

**Figure 1:**
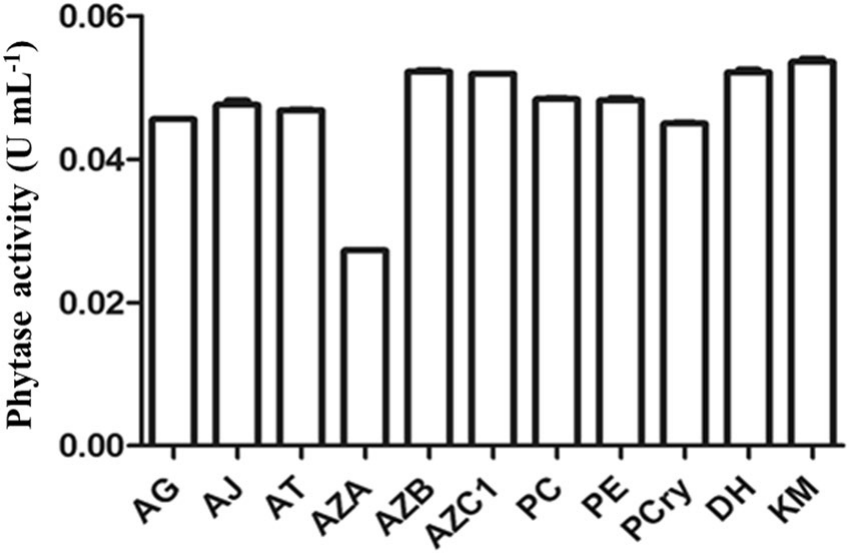
Phytase activity in microbial extracts of *AG: Aspergillus glaucus, AJ: A. japonicus, AT: A. terreus, AZA: Acremonium zeae A, AZB: A. zeae B, AZC1: A. zeae C1, PC: Penicillium chrysogenum, PE: P. expansum, PC: P. citrinum, DH: Debaryomyces hanseniie and KM: Kluyveromyces marxianus* cultured in liquid medium. The bars correspond to the standard deviations of the means (n = 3).

All the microorganisms studied produced phytases and their activities did not differ among the fungi *Aspergillus glaucus*, *A. japonicus*, *A. terreus*, *Acremonium zeae B*, *A. zeae C1*, *Debaryomyces hansenii* and *Kluyveromyces marxianus* (Fig. 1). The fungi *A. zeae* B and *K. marxianus*, with activities of 0.05 U mL^−1^ and 0.06 U mL^−1^, respectively, were selected for further analysis.

Microorganisms are the main source of enzymes and other compounds of biotechnological interest^10^. The production of phytases by these fungi confirms previous results found for *Aspergillus* spp.^11–14^ and *Penicillium* spp.^5,15,16^]. Fungi belonging to the genus *Acremonium*, relatively unknown for phytase production, presented an yield similar to other widely known fungi^11,15^. The species of this genus are endophytic^17^ and can produce compounds of biotechnological interest, mainly antibiotics^18,19^. Other endophytic fungi were already described as phytase producers, including some species of the genus *Mucosodor* sp.^20^ and *Rhizoctonia* sp.^21^. Thus, considering the potential of this genus for industrial phytase production, the fungus *A. zeae* B was selected for further evaluations. The yeast *K. marxianus* also presents good performance for large scale fermentations^22^ and the production of various compounds, such as bioethanol^23,24^, β-galactosidase^25,26^ and probiotics^27,28^. Therefore, it was selected as a model strain for phytase production.

### Determination of conditions for enzyme production by the microorganisms

#### Substrate

**Figure 2:**
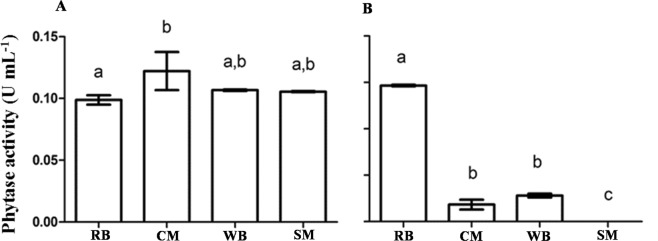
Phytase production by *Acremonium zeae* B (**A**) and *Kluyveromyces marxianus* (**B**) in media containing rice bran (RB), corn meal (CM), wheat bran (WB) or soybean meal (SM). *A. zeae* was grown in semi-solid medium containing 20% (m/v) of the substrate for 120 h and *K. marxianus* in liquid medium containing 1.0% (w/v) of each substrate for 48 h. The bars correspond to the standard deviations of the means (n = 3). Equal letters do not differ at 95% confidence by the Tukey test.

**Figure 3:**
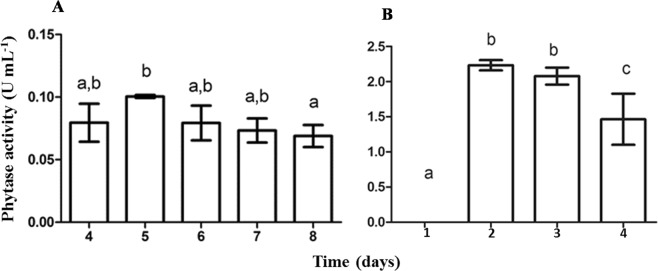
Production of phytase over the incubation time by the fungus *Acremonium zeae* B (**A**) from corn meal and the yeast *Kluyveromyces marxianus* (**B**) from rice bran, via solid-state fermentation and submerged fermentation, respectively. The bars correspond to the standard deviations of the means (n = 3). Equal letters do not differ at 95% confidence by the Tukey test.

The fungus *A. zeae* B produced phytase from different substrates (Fig. 2A), but the highest productivity was achieved when corn meal (CM) was added to the medium, with the maximum activity obtained at 120 h (Fig. 3A). After this time, the total activity decreased. In media containing wheat bran (WB) and soybean meal (SM), the maximum activity occurred at 144 h and was lower then that using CM (data not shown). On the other hand, the yeast *K. marxianus* produced phytase only in rice bran (RB) (Fig. 2B) at 48 h. The total phytase activity in the yeast extract was higher than that observed in the extract of the fungus, and the time required for achieving higher activity was twice as low (Fig. 3B). This means that the productivity of this yeast (3.525 U day^−1^) was 10-fold higher compared to that of the fungus (0.3 U day^−1^).

The use of agroindustrial byproducts as substrates for the production of enzymes has become the main goal of various studies due to the low cost of these raw materials^11^, which could enable the industrial production of several enzymes. The high phytase productivity by the fungus *A. zeae B* in CM and WB indicated the possibility of using such low-cost byproducts as alternative sources for phytase production. The substrate selected for phytase production by the fungus *A. zeae* B (CM) suggests that phytase production is not only related to the percentage of phytic acid in the medium, since CM has less phytic acid than WB. One of the explanations relies on the fact that this fungus was isolated from CM. Thus, it was adapted and effectively assimilated the nutrients from this agroindustrial byproduct. The decreased phytase activity with further incubation may indicate the action of proteases, which hydrolyze proteins in the medium to provide amino acids for the microbial population.

On the other hand, the higher productivity of the yeast *K. marxianus* in RB (0.14 U mL^−1^) was probably stimulated by the high concentration of phytic acid (5.1 to 8.6%) in this substrate^29^. High concentrations of phosphate in the culture medium down regulated phytase synthesis by *Candida* sp.^30^. This could explain the absence of phytase activity in the culture of *K. marxianus* in RB until 24 h. The lower phytase production by the yeast when cultivated in the other agroindustrial byproducts (CM, SM and WB) suggests a limited use of nutrients by this yeast, or the repression of the enzyme synthesis by compounds from these substrates.

The yield of enzymes from agroindustrial byproducts varies and depends on the species of the cultured microorganism. For example, *Humicola nigrescens*^31^ and *Schizophyllum commune*^32^ yielded higher amounts of phytase in WB, *P. funiculosum* NRC467^7^ using broad bean and *Penicillium purpurogenum GE1* when cultivated in corn cob^11^.

#### Effect of temperature, pH and substrate concentration on phytase production

**Figure 4:**
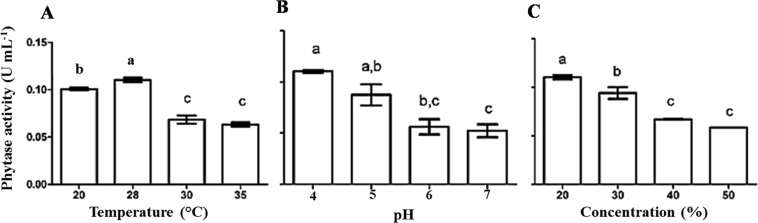
Phytase production by *Acremonium zeae* B via solid state fermentation under different conditions of temperature (**A**), pH (**B**) and substrate concentration (**C**). The fixed parameters for each experiment were, when applicable, temperature of 28 °C, pH 4.0 and 10% (m/v) of corn meal. The bars correspond to the standard deviations of the means (n = 3). Equal letters do not differ at 95% confidence by the Tukey test.

The fungus *A. zeae* B generated extracts with higher phytase activity when incubated at 28 °C, pH 4.0 and 20% (w/v) of CM (Fig. 4). Substrate concentrations higher than 20% (w/ v) decreased phytase production. Increased substrate concentration reduced the amount of water available for the microorganism, which limited enzyme production.

Temperature is one of the main parameters affecting the microbial growth and production of metabolites^31,33^. The higher phytase production by the fungus at 28 °C can be related to the narrow range of temperature considered ideal for its growth (between 20 and 25 °C), ceasing at 45 °C^34^.

**Figure 5:**
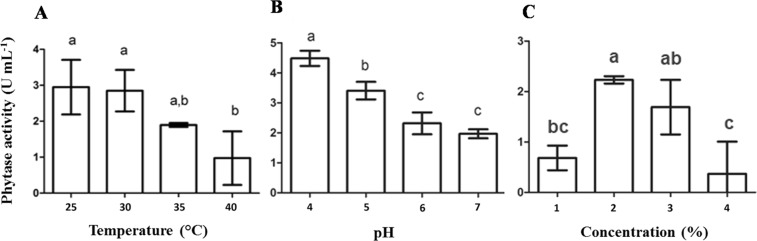
Phytase production by *Kluyveromyces marxianus* via submerged fermentation under different conditions of temperature, pH (**B**) and substrate concentration (**C**). The fixed parameters for each experiment were, when applicable, temperature of 28 °C, pH 5.8 and 1% (m/v) of rice brain. The bars correspond to the standard deviations of the means (n = 3). Equal letters do not differ.

The optimum conditions for phytase production by the yeast were different from those for the fungus. *K. marxianus* produced higher amounts of the enzyme at 25–30 °C, pH 4.0 and 2% (w/v) substrate. These conditions were also significantly distinct from those originally used for the cultivation of this yeast (1% (w/v) substrate, pH 5.8 and 28 °C) (Fig. 5).

The combination of the best conditions for the yeast allowed producing 101.25 U day^−1^ of phytases, an index 30-fold higher than the original. This result was achieved using a low-cost medium and mild processing conditions, ideal for industrial processes.

### Characterization of the phytase activity in the extracts

**Figure 6:**
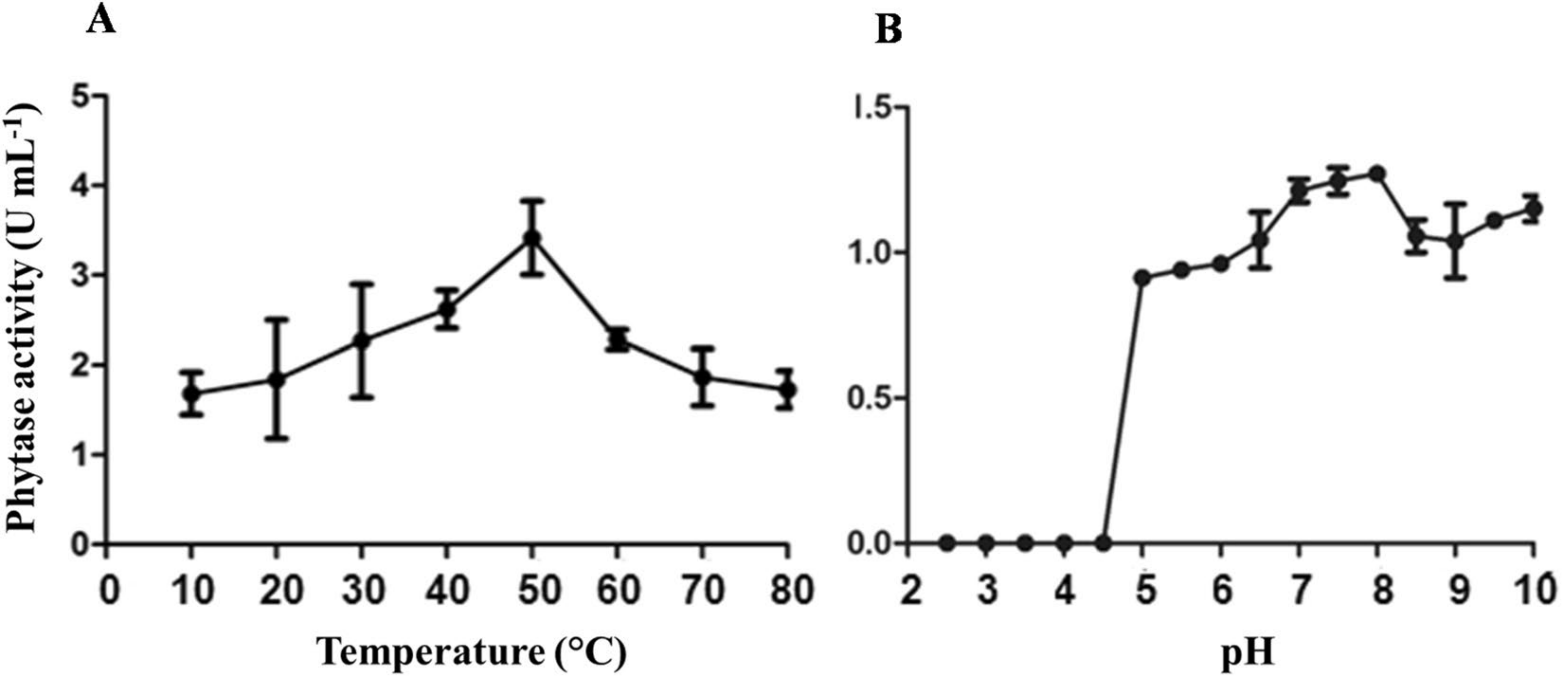
Phytase activity in *Acremonium zeae* B extracts at different temperatures (**A**) and pH values (**B**). The bars correspond to the standard deviations of the means (n = 3).

The ideal conditions for phytase activity in the extracts of *A. zeae* B were 50 °C and neutral pH. At acidic pH values (≤ 4.5), the extract completely lost phytase activity (Fig. 6).

The conditions for phytase activity in *A. zeae* B extracts (50 °C and neutral pH) are common for microbial phytases^31,35^. However, the pH of the gastrointestinal tract of pigs ranges from 2.0 to 4.5^36^. Therefore, this extract would probably completely halt the activity of this enzyme in that environment. Some alternatives could allow the use of this extract in feed for monogastric animals. The use of encapsulating agents, for example, would protect the enzymes against the stomach pH and allow their release in the intestine, where the phosphorus is absorbed^37^. Another alternative relies on the hydrolysis of the phytate from raw materials and feed supplementation with free phosphorus.

**Figure 7:**
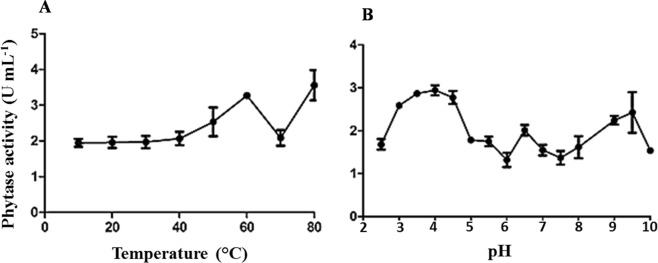
Phytase activity in *Kluyveromyces marxianus* extracts at different temperatures (**A**) and pH values (**B**). The bars correspond to the standard deviations of the means (n = 3).

On the other hand, the extract obtained from *K. marxianus* presented peaks of maximum activity of phytase at 60 and 80 °C. Besides, it was stable throughout the pH range tested (2.5 to 10.0). The highest activity was obtained at pH 4.0 and the smallest, close to the neutral pH (Fig. 7).

The existence of two peaks of optimum activity, associated to the high stability observed for phytase activity in the *K. marxianus* extract, suggest the presence of more than one enzyme. The ability of the *K. marxianus* extract to maintain hydrolytic activity from pH 2.0 to 6.5, which is necessary to facilitate degradation of phytate in the salivary glands, stomach and upper duodenum of monogastric animals^14^, would allow a prolonged degradation of phytate throughout the digestive system of these animals and an efficient catalytic action in the stomach^4^. In general, these enzymes are relatively unstable in media with pH higher than 7.5 and lower than 3.0^14^, with the exception of some acidic phytases with optimal pH of 1.5–2.5^37–39^. The ideal conditions for enzyme activity depend on the source from which they are obtained and the environment in which they act^37^. Bacterial phytases are usually neutral to alkaline, while fungi phytase optimum pH ranges from 2.5 to 6.0^11^.

**Figure 8:**
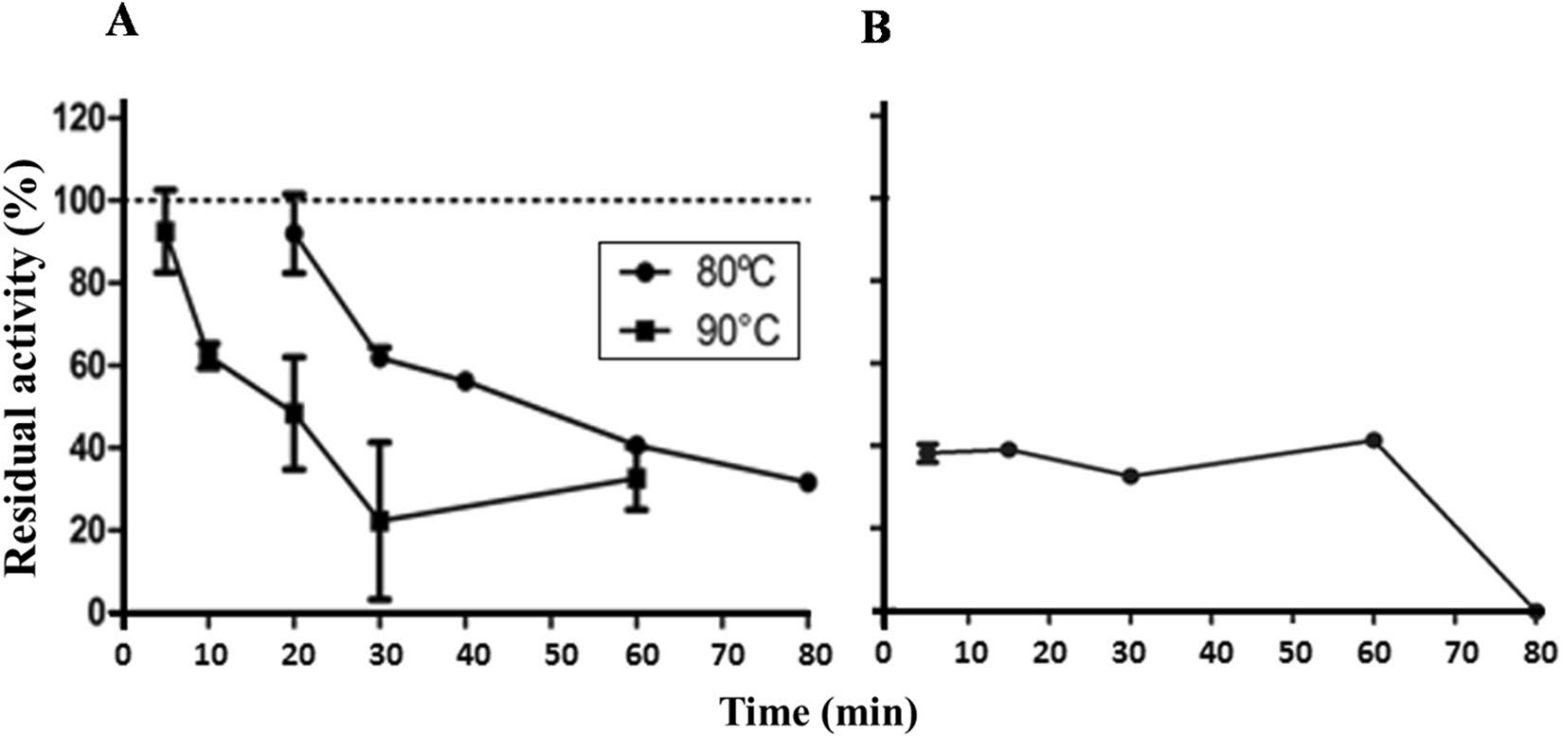
Thermostability at 80 and 90 °C in *Acremonium zeae* B extracts (**A**) and at 80 °C in *Kluyveromyces marxianus* extracts (**B**). The bars correspond to the standard deviations of the means (n = 3). Data expressed in terms of residual activity consider the activity value of the extract at zero incubation time as 100%.

Despite the high optimum temperatures for phytase activity presented by the yeast extract, the thermostabiliy of these enzymes was lower than that of the *A. zeae* B extract. The *A. zeae* B extract maintained its initial phytase activity even after 10 min at 80 °C or 5 min at 90 °C (Fig. 8).

The thermal stability is an important factor for the use of phytases in animal feed. During feed production, the extract can be exposed to high temperatures^40^ such as 80 °C for a maximum of 2 min. The thermal stability of phytases in the *A. zeae* B extract exceeded that presented by the commercial enzyme Natuphos® (BASF) (data not shown), used in diet manufacturing. This enzyme maintained only 60% of this original activity when incubated for 5 min at 80 °C, and less than 20% after 15 min. The activity of this enzyme was further completely inhibited after 15 min at 90 °C. A purified phytase of *A. flavus* ITCC 6720 presented relative activity of 32, 10 and 0% when incubated at 50 °C for 10, 20 and 30 min, respectively^41^.

The *K. marxianus* extract presented lower thermal stability. It maintained only 40% of its original activity after 80 °C for 20 min and was completely inhibited when subjected to the temperature of 90 °C (Fig. 8). Tests performed at 60 and 70 °C showed that the *K. marxianus* extract was able to maintain up to 80 and 60% of its original activity after 120 min of incubation, respectively (data not shown). Therefore, it is regarded as of moderate stability to thermal processing conditions. Phytases generally exhibit increased activity at temperatures of 50 to 70 °C^14^, above which they denature and lose their activity. The total loss of activity of a purified phytase of *Aspergillus ficcum* NTG-23 occurred at 80 °C for 10 min^42^. A purified phytase from *Rhizopus oligosporus* ATCC 22959 maintained only 20% of its residual activity after 5 min at 80 °C^5^.

Considering the other characteristics of the enzymes present in the *K. marxianus* extract, its use could still be sufficient for feed production, since the pelletizing process is carried out under conditions less restrictive than those tested in this work. Moreover, some feed production processes do not comprise this step at all. Some additional alternatives include enzymatic immobilization, to increase enzyme stability^43^ or the addition of viable yeasts to moist rations, where it would produce phytase and other beneficial compounds^44^.

Metal ions also significantly affected the phytase activity in the extracts of *K. marxianus* and *A. zeae* B. The *A. zeae* B extract was strongly inhibited by CaCO_3_, Ag_2_SO_4_, FeSO_4_, NH_4_Cl_2_ and CuSO_4_ and, to a lesser extent, by ZnSO_4_. The opposite occurred with the enzymes in the *K. marxianus* extract, whose activity increased until 3.9-fold in the presence of Ca^2+^ ions, regardless of the salt from which the ion was originated. The presence of 2 mM CuSO_4_ in this extract also increased enzyme activity by 3-fold. AgSO_4_, MgCl_2_ and NH_4_Cl_2_ also increased the activity in the extract, while only ZnSO_4_ and FeSO_4_ caused partial enzymatic inhibition (Table 1). The effect of ions on phytase activity varies, but the inhibition by Zn^2+^ and Cu^2+^, as observed in the *A. zeae* B extract, has been previously observed^45^. A purified phytase from *A. niger* UFV-1 was inhibited, at different levels, by Mg^2+^, Ca^2+^, Mn^2+^, Cu^2+^ and Zn^2+^ at 5 mM concentration^37^.

**Table 1:** Effect of ions on phytase activity in *Acremonium zeae* B and *Kluyveromyces marxianus* extracts.

Salt	*Acremonium zeae* B		*Kluyveromyces marxianus*	
	2 mM	5 mM	2 mM	5 mM
CaCl_2_	89.5 ± 0.04	115.0 ± 4.6	116.0 ± 2.8	273.0 ± 3.1
CaCO_3_	91.1 ± 0.0	ND	144.0 ± 3.1	203.0 ± 1.8
AgSO_4_	79.0 ± 0.3	ND	116.7 ± 12.1	289.0 ± 20.6
MgCl_2_	95.6 ± 1.0	119.2 ± 1.0	217.0 ± 3.87	268.0 ± 4.6
ZnSO_4_	62.0 ± 0.0	49.8 ± 0.06	60.6 ± 1.4	32.8 ± 2.5
CuSO_4_	98.0 ± 0.0	ND	310.0 ± 31.3	135.6 ± 3.0
CaSO_4_	99.3 ± 0.02	123.0 ± 0.1	204.0 ± 0.5	387.0 ± 9.5
NH_4_Cl_2_	98.0 ± 0.0	2.0 ± 0.0	127.0 ± 4.8	238.0 ± 14.1
FeSO_4_	97.0 ± 0.3	ND	96.0 ± 0.3	52.0 ± 1.0

Data are expressed in terms of relative activity considering 100% the activity in the extract without addition of ions.

ND = activity not detected in the condition tested. ±standard deviation corresponding to the mean of triplicates.

The increased activity in *K. marxianus* extract in the presence of Ca^2+^, CuSO_4_, AgSO_4_, MgCl_2_ and NH_4_Cl_2_, and the maintenance of phytase activity in the presence of most salts show the stability of this extract to metallic ions, which favors its use, since ions are commonly present in animal feed^37^. The inhibition of the phytase activity in the *A. zeae* B extract by most ions highlights the sensitivity of this enzyme to varied conditions.

When evaluated together, the high stability of the enzymatic extract of the yeast *K. marxianus* at different pH values, temperature range and ions, associated to the high productivity of this microorganism, are promising characteristics for the use of this extract in feed production. Moreover, the productivity of this microorganism was superior to that of the fungus. This evidences the feasibility of the commercial use of this extract in the short term, with low production costs achieved with the use of byproducts as substrates, mild process conditions and the fact that the enzyme from the extract demands no purification for being used.

## Conclusions

The use of agroindustrial byproducts as substrates successfully induced phytase production by the yeast *K. marxianus* and the endophytic fungus *A. zeae* B. It could be an alternative to reduce the costs of phytase production for addition to animal feed. The high stability at different pH values, temperature range and ions of the phytases in the *K. marxianus* extract, associated with shorter generation time and high productivity, makes this yeast a potential candidate for industrial phytase production.
